# Supplementary material for: Interference suppression techniques for OPM-based MEG: Opportunities and challenges
Source: Neuroimage. 2022 Feb 15;247:118834. doi: 10.1016/j.neuroimage.2021.118834 (PMC8803550; doi:10.1016/j.neuroimage.2021.118834)
Supplement: Supplementary file 1 [file mmc1.docx]

**Supplementary Materials**

**Article**: Interference suppression techniques for OPM-based MEG:

Opportunities and challenges

**Authors:** Robert A. Seymour, Nicholas Alexander, Stephanie Mellor, George C. O’Neill, Tim M. Tierney, Gareth R. Barnes, Eleanor A. Maguire

***Demonstrating the effect of filtering on OPM-based MEG movement artefacts:***

Below we plot an example OPM movement-related artefact from the first example data analysis tutorial (Section 5.1, main text), and the effect of some of the filtering techniques. The interested reader can also download the raw data to visualise the artefacts and try out the filtering approaches for themselves.


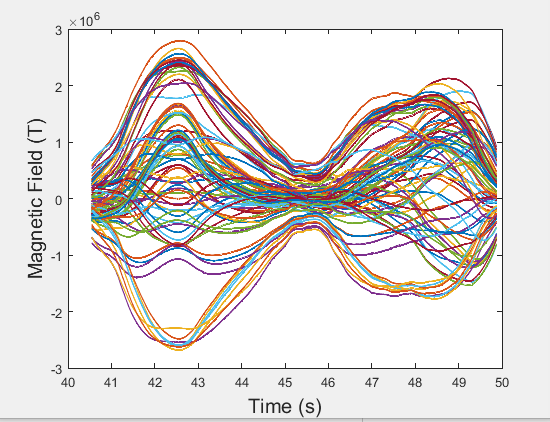


**Fig. S1.** Ten s-long sample of raw data before high-pass filtering.


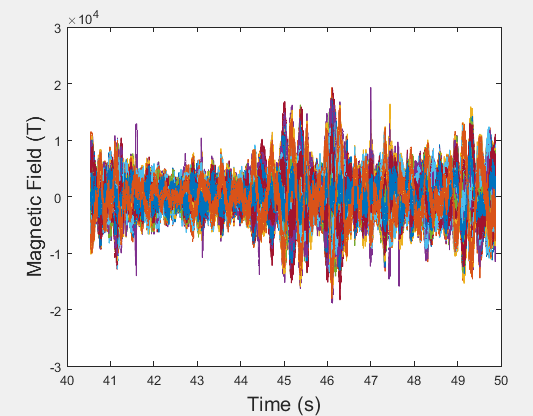


**Fig. S2.** Ten s-long sample of data after high-pass filtering at 2 Hz, using same parameters as discussed in the main text. Note the reduction in low-frequency drifts and change in scale of the y-axis.


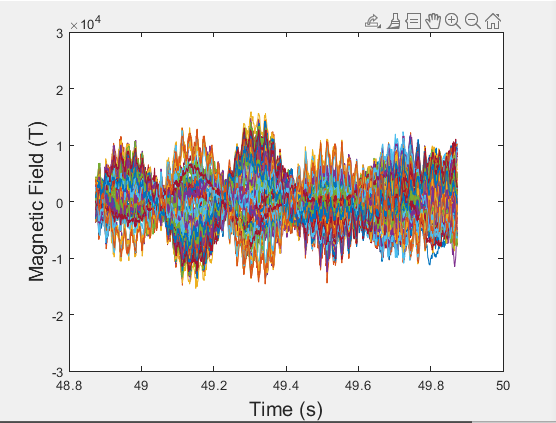


**Fig. S3.** 1.1 s-long sample of the same data after high-pass filtering at 2Hz, but before low-pass filtering.


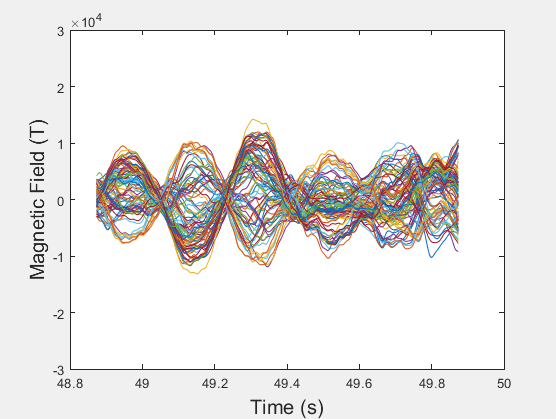


**Fig. S4.** 1.1 s-long sample of data after high-pass filtering at 2Hz and low-pass filtering at 40Hz using the same parameters as discussed in the main text. Note the reduction in high-frequency AC activity.
